# The COVID-19 pandemic: time for medical teachers and students to overcome grief

**DOI:** 10.6061/clinics/2020/e2206

**Published:** 2020-07-27

**Authors:** Patricia Tempski, Arthur H. Danila, Fernanda M. Arantes-Costa, Marina A.M. Siqueira, Matheus B. Torsani, Milton A. Martins

**Affiliations:** Centro de Desenvolvimento de Educacao Medica e Departamento de Clinica Medica, Faculdade de Medicina FMUSP, Universidade de Sao Paulo, Sao Paulo, SP, BR

As health professionals and medical educators, we have had a huge challenge in the past few months to adapt our professional lives to the COVID-19 pandemic. This has been the most important and necessary modification of our professional activities in the last few decades (1,2). Suddenly, much of what we used to do as teachers has had to be interrupted and/or modified, and we have had to exercise flexibility and creativity, adapting medical education to virtual environments. The COVID-19 pandemic has had a profound effect on medical education, and these times may forever change how future physicians are educated. In Brazil, as in many other countries, first-year medical students have to stay at home and all teaching and learning strategies have had to be changed to distance learning.

In our medical school, faculty and medical students have received the support of the Center for Development of Medical Education, which has helped teachers in adapting their educational programs and student assessments to the new reality of distance learning. In the last few weeks, we have observed faculty and students and their different actions, behaviors, and difficulties during this urgent transition. Although we have all experienced losses of freedom and security concerning our health and the future, we noticed differences in both the time people take to adapt as well as in their responses to the challenge of change in their roles as medical educators during the COVID-19 pandemic.

We propose the model of Kubler-Ross (3) to explain the different reactions and opinions of faculty and students concerning the need for change in medical teaching strategies during the COVID-19 pandemic.

The psychiatrist Elisabeth Kubler-Ross postulated that there are five stages of emotions experienced by terminally ill patients or people who have lost a loved one: denial, anger, bargaining, depression, and acceptance (4). The Kubler-Ross model of grief can be useful in explaining the reactions of many of our colleagues and students regarding the transition to emergency remote learning.

In the first weeks of quarantine, teachers’ comments were often indicative of denial: “I will not change my lectures to the virtual environment because this situation will quickly return to normal,” or in the case of medical students: “This pandemic is not as severe as people say it is; we can wait and restart everything within a few weeks.”

After two or three weeks of social isolation, we noticed several manifestations of anger, mainly in first-year medical students who had just started medical school and had to stay at home, as well as final-year medical students who had to stop their clerkships: “It is impossible to learn medicine when everything is virtual,” “There will be a substantial loss in the quality of my training as a physician.”

At the beginning of the second month of social isolation, with the increase in the number of COVID-19 cases and the fact that the return to medical school would take many months, we observed some teachers and students with behaviors consistent with bargaining. They started to negotiate what they could do after the end of the isolation period, in terms of classes, other activities, and assessments. Some medical students started to negotiate whether their voluntary activities could be considered equivalent to some clerkships to avoid delays in finishing medical school.

The city of Sao Paulo became the epicenter of the pandemic in Brazil, with an increasing number of COVID-19 cases and deaths. Teachers and students began to manifest depression due to the stress involved in caring for people with COVID-19, the personal risk involved in this caring, the social isolation, and insecurity about one’s personal and professional future.

Now, we observe that many people have begun to realize that there are gains and not only losses in this period, and acceptance has become more common. Many faculty and students have started to talk about the positive aspects of the COVID-19 pandemic: volunteering, mentoring, service to community, altruism, building professional identity, defining what is really important in the objectives and content of the medical program, and inter-professional education.

The emotional state of the teacher interferes with his/her teaching activities and students’ learning and his/her motivation to learn and apply new technologies or to resist any innovation. Medical schools must provide emotional and pedagogical support to their faculty and students, considering the challenges of the COVID-19 times.

Many educators are not adequately acquainted with the increasing technological possibilities of the 21^st^ century, being digital immigrants, whereas the students are digital natives (5). Many teachers made mistakes trying to do their best and students recognized their efforts, showing empathy toward their teachers’ dedication. Medical students helped their teachers, and strong collaboration developed between teachers and students, sometimes stronger than before the pandemic. The number of teachers who decided to participate in faculty development activities was higher than before.

However, those who have changed, adapted, and tried to do something to maintain the educational process have glimpsed the future, experiencing a different way of teaching and learning. Teachers and students who have experienced the loss of traditional medical training, even if it was temporary, may or may not have experienced the phases of grief, but they have certainly emerged from the experience wiser and more resilient, with new skills.

The use of the Kubler-Ross model has been criticized since it has not always been empirically demonstrated and many patients do not show all these stages or exhibit elements of more than one stage concurrently. This might also be true regarding the feelings of teachers and students in the COVID-19 pandemic, that only some people have gone through all stages and many have experienced feelings that correspond with more than one stage of the Kubler-Ross model. How each person experiences and deals with difficulties depends on his/her resilience (6).

However, our reflection is useful for faculty and students to understand that it is possible to accept the challenges of the COVID-19 pandemic as an opportunity to become better doctors.

## AUTHOR CONTRIBUTIONS

All authors participated in the discussions and writing of the manuscript and reviewed the manuscript in its final form.
